# Awareness of Heightened Sexual and Behavioral Vulnerability as a Trigger for PrEP Resumption Among Adolescent Girls and Young Women in East and Southern Africa

**DOI:** 10.1007/s11904-023-00680-y

**Published:** 2023-12-05

**Authors:** Krishnaveni Reddy, Thesla Palanee-Phillips, Renee Heffron

**Affiliations:** 1 Wits RHI, Faculty of Health Sciences, University of the Witwatersrand, Johannesburg, South Africa; 2 University of Washington, Seattle, WA USA; 3 University of Alabama at Birmingham, Birmingham, AL USA

**Keywords:** Oral PrEP, HIV vulnerability, Adolescents, Restart

## Abstract

**Purpose of Review:**

East and Southern Africa are the epicenter of the HIV epidemic. High HIV incidence rates among adolescent girls and young women (AGYW) remain stable over the last decade despite access to daily oral PrEP. Some settings have experienced high PrEP uptake among AGYW; however, discontinuation has been high. This review sought to understand drivers of PrEP discontinuation in this population in order to identify potential mechanisms to facilitate PrEP restart and optimize PrEP use.

**Recent Findings:**

Drivers of PrEP discontinuation included low perceived HIV acquisition risk, PrEP-associated side effects, pill burden, family/sexual partner disapproval, lack of/intermittent sexual activity, PrEP use stigma, fear of intimate partner violence, misinformation about long-term PrEP use, and limited/inconsistent access to PrEP.

**Summary:**

The most frequently reported driver of PrEP discontinuation was low perceived HIV acquisition risk. This indicates that innovative interventions to help AGYW recognize their HIV risk and make informed decisions about PrEP use are urgently needed.

## Introduction

East and Southern Africa (ESA) remain the epicenter of the HIV epidemic with 20.7 million people living with HIV (representing 55% of the number of people living with HIV worldwide even though this region accounts for < 8% of the world’s population) [1–3]. ESA includes 15 of the top 28 countries with HIV infections globally, 8 of which (Botswana, Eswatini, Lesotho, Mozambique, Namibia, South Africa, Zambia, and Zimbabwe) have some of the world’s highest HIV prevalence rates, ranging from 11% to over 26% among adults [1]. Cisgender heterosexual women comprise one of the most affected populations in this region with adult women comprising 3 in 5 new HIV infections in the region in 2020 and adolescent girls being 2.6 times more likely to acquire HIV than their male peers [1].

These high rates of new HIV infection have continued to occur despite the substantial investment that has been made in ESA over the past decade to scale-up education and access to HIV testing, implement universal testing and treatment of HIV, and roll out oral pre-exposure prophylaxis (PrEP) as an efficacious user-controlled HIV prevention strategy [4, 5]. Daily oral PrEP consisting of tenofovir disoproxil fumarate/emtricitabine (TDF/FTC) in a single fixed-dose combination pill has been the flagship PrEP regimen [6]. It was approved by the US Food and Drug Administration (FDA) for HIV prevention by uninfected individuals in July 2012 [7] and was recommended by the World Health Organization (WHO) to be offered to people at substantial risk of HIV infection as part of comprehensive prevention in September 2015 [8]. South Africa became the first country in Africa to approve oral PrEP with its drug regulatory authority providing approval in November 2015 [9] and followed closely by Kenya in December 2015 [10]. Subsequently, drug-regulatory authorities in ten other African countries have approved a formal indication for TDF-based formulations as HIV PrEP, and national policies in nine countries in Africa have incorporated PrEP as part of a prevention strategy [11].

Since 2012, additional PrEP products have advanced significantly through stages of product development and testing. Beginning in July 2020, regulatory authorities, including the European Medicines Agency (EMA) and the World Health Organization, issued approvals for the dapivirine vaginal ring, a monthly-replaced silicone matrix ring containing the novel antiretroviral dapivirine, as an additional prevention option for adult cisgender women at substantial risk of HIV infection [12, 13]. Reviews and approvals by national bodies in South Africa, Zimbabwe, Kenya, and others have taken place since 2021 [14, 15]. Unlike oral PrEP though, the dapivirine vaginal ring is only approved for women ≥ 18 years at this time. In 2020, the first efficacy data for a 2-monthly cabotegravir injectable suspension relative to daily oral TDF/FTC were announced with superiority demonstrated across populations [16, 17]. Regulatory approvals in the USA followed in late 2021 for use as HIV PrEP in at-risk adults and adolescents weighing at least 35 kgs [18]. Zimbabwe was the first country in Africa to approve injectable cabotegravir as PrEP [19] followed by South Africa and others [20]. Development and testing of other novel longer-acting agents are in progress, including some that aim to bundle prevention of pregnancy or other sexually transmitted infections (STIs), alongside their planned HIV prevention benefit [6].

As the HIV prevention field advances to develop new products and increase HIV prevention coverage, understanding drivers of discontinuation is paramount to guide product developers and local providers to be more attentive to end-user preferences and barriers to use of available effective modalities and to identify mechanisms that may prompt PrEP restart and optimize PrEP use.

## PrEP Use Among Adolescent Girls and Young Women (AGYW) in ESA

AGYW are disproportionately impacted by the HIV epidemic, having several behavioral, biological, and socioeconomic characteristics that cause them to be vulnerable to contracting HIV and contribute to their high HIV incidence rates [21]. These include but are not limited to the following: early sexual debut, engaging in age-disparate relationships that are associated with inconsistent condom use and transactional sex, financial insecurity and gender inequalities that limit their agency to negotiate sex and condom use in order to maintain relationship security and avoid violence from their sexual partners, and high prevalence of other STIs which predispose them to a higher risk of HIV acquisition [22–24]. They are also biologically more susceptible to HIV than young men due to the comparatively larger surface area of the cervix and vaginal mucosa and differences in the mucosal immunology [25].

Since 2015 when oral PrEP was approved in these regions [9–11], several oral PrEP demonstration projects and research studies have targeted AGYW (Table 1). Synthesis of this work in research clinics and STI/HIV clinics shows that AGYW have been willing to start using PrEP with an initial prescription and bottle of pills (estimates ranging from 55 to 100% with highest uptake seeming to occur in programs offering PrEP initiation in safe space settings). One exception is the public family planning clinic setting in Kenya that hosted PrIYA where only 22% of 1271 women screened initiated PrEP [26]. In that program, a substantial proportion of women had partners of unknown HIV status and felt they needed to consult their male partners before they could consider PrEP; however, uptake among women whose partner was known to have HIV was 94% demonstrating that women with known exposure recognized the benefits of PrEP and were willing to use it. When asked about factors motivating uptake of oral PrEP, AGYW responses have included the following: increased autonomy over their sexual health independent of sexual partners’ knowledge or approval, desire for HIV protection from sexual partner/s with multiple concurrent partnerships, unknown HIV status, and/or low condom use (either to avoid hostile condom negotiations, to please a long-term partner, or due to peer pressure), to protect themselves from HIV when engaging in transactional sex or sex under the influence of alcohol or when exposed to sexual violence in their communities, and to reduce HIV-related anxiety [27, 28].

**Table 1:** AGYW oral PrEP uptake and persistence in oral PrEP clinical trials and demonstration projects in ESA

Clinical trials/demo projects	Location	Duration	Participant age (years)	Number enrolled	Initial oral PrEP uptake	Persistence	Adherence by drug level measurement (TFV-DP levels via DBS)			
							1 month	3 months	6 months	12 months
HPTN 082/HERS [33]	South Africa (Cape Town, Johannesburg), Zimbabwe (Harare)	Oct 2016–Oct 2018	16–25	451 Harare, 148 Cape Town, 141 Johannesburg, 162 *(Clinical trial sites)*	95%	12 months: 55%	N/A	84%	57%	31%
MPYA [34, 35]	Kenya (Thika, Kisumu)	Dec 2016–Mar 2020	18–24	348 *(Adolescent friendly research clinics)*	100%		Electronically monitored adherence declined from 65% (month 0–1) to 15% (months 22–24). Electronically monitored adherence data were available for 150 (85%) of 177 TFV-DP samples with 67% concordance seen between the 2 measures			
DREAMS PrEP Program [30]	Kenya (Kisumu, Homa Bay)	Mar 2017–Dec 2017	15–24 years	1259 Kisumu, 572 Homa Bay, 687 *(Safe space settings)*	100%	1 month: 57% 2 months: 46% 3 months: 37%	Not done	Not done	Not done	Not done
POWER [27, 36]	Kenya (Kisumu), South Africa (Cape Town Johannesburg)	Jun 2017–Sep 2020	16–25	2550 Kisumu, 1000 *(Family planning clinics)* Cape Town, 787 (*Youth and primary healthcare clinics)* Johannesburg, 763) (*Youth and primary healthcare clinics)*	94%	31% returned for the first refill, 20% persisted without ≥ 15 day gap in refills, 14% stopped and restarted after a gap of ≥ 15 days	Among 1156 participants eligible for 3 and 6 month visits, 193 (16.7%) specimens were selected and indicated ~ 47% took an average of ≥ 4 doses/week			
DREAMS, Namibian Ministry of Health and Social Services [31]	Namibia (Khomas)	Oct 2017–Sep 2019	15–24	1994 372 Facility Model (*Public health facilities)* 302 Community Concierge Model *(Safe space settings)* and 1320 *Hybrid community-clinic model (Initiation at safe spaces and referral to public facility for refills/follow-up)*	100%	Facility Model 1 month: 36.8% 3 months: 26.7% Community Concierge Model 1 month: 41.2% 3 months: 34.9% Hybrid community-clinic model 1 month: 6.2% 3 months: 4.8%	Not done	Not done	Not done	Not done
PrIYA [26, 40]	Kenya (Kisumu)	Nov 2017–June 2018	15 to 45 (PrEP offered during antenatal/breastfeeding period)	1271* (Family Planning Clinics)*	22%	Month 1: 41%, Month 3: 24%, Month 6: 15%	Not done	Not done	Not done	Not done
TB HIV Care [32]	South Africa	2018–2020	15–35 years or older	28,100* (Integrated health services clinics)*	55%	1 month: 38%	Not done	Not done	Not done	Not done

Behaviors necessary for sustained HIV protection, such as persistence and adherence, are however frequently cited challenges for AGYW [29, 30]. Overall, PrEP persistence decreased over time across PrEP programs and studies of AGYW (Table 1). Data from the DREAMS PrEP Program in Kenya, one of the largest real world demonstration projects, reported a median PrEP persistence time of 56 days among AGYW who initiated PrEP with the proportions of AGYW who persisted in the PrEP program at 1, 2, and 3 month(s) after PrEP initiation being 57%, 46%, and 37% respectively [30]. Further to this, data collected from DREAMS PrEP service delivery in Namibia’s Khomas Region (372 (18.7%) AGYW through a facility model, 302 (15.1%) through a community model, and 1320 (66.2%) through a hybrid model) showed PrEP persistence at 1 and 3 months to be 36.8% and 26.7% in the facility model, 41.2% and 34.9% in the community model, and 6.2% and 4.8% in the hybrid model [31]. The TB HIV Care (THC) PrEP program initiated 28100 AGYW on PrEP between 2018 and 2020. The AGYW included accessed health services from THC (e.g., HIV testing, sexual and reproductive health services) and were at greater risk of HIV acquisition than AGYW more broadly, with possibility of age-disparate relationships, multiple partnerships, transactional sex, or unstable home environments and/or school attendance. Results from this program indicated that about 38% of participants remained on PrEP at 1 month. PrEP stop and restart were common with early missed visits and inconsistent, but ongoing use [32].

In studies in research clinics, persistence appeared somewhat better than public clinic settings. In the HPTN 082/HERS study in South Africa and Zimbabwe, oral PrEP uptake among young women aged 16–25 years was 95% (*N* = 427), and 55% had uninterrupted PrEP refills through 12 months. Of those with dried blood spots (DBS) samples to measure oral PrEP adherence, 84% had detectable tenofovir-diphosphate (TFV-DP) levels at month 3 that declined as the study progressed (57% at month 6 and 31% at month 12) [33]. The MPYA study in Kenya, determining the impact of SMS reminders on PrEP adherence, enrolled 348 AGYW aged 18–24. Adherence was measured with pharmacy refill and real-time electronic monitoring, plus tenofovir diphosphate levels in 15% of participants. Pharmacy refills steadily declined from 100% (month 0–1) to 54% (months 22–24) and average electronically monitored adherence similarly declined from 65% (month 0–1) to 15% (months 22–24) with moderately high concordance with TFV-DP levels (67%) [34, 35]. In the POWER study in Kenya and South Africa set in diverse settings (research clinic, mobile units, public family planning), 2397 AGYW (94%) initiated PrEP and only 749 (31%) had a refill at 1 month. Of AGYW who reached 6 months or more of post-PrEP initiation follow-up, there was considerable lack of persistence with 128/646 (20%) persisting with PrEP for 6 months and great variation by site (8% to 38%). Interestingly, 14% of participants with a gap in PrEP use restarted PrEP at some point during follow-up. Among a small subsample (16.7%), intracellular TFV-DP levels indicated that 19.0% had taken PrEP daily, 28.2% an average of 4–6 doses per week, 5.1% an average of 2–3 doses per week, 19.5% an average of 1 dose per week, and 28.2% had TFV-DP levels below the limit of quantification [36]. Additionally, these outcomes demonstrate that low persistence and low adherence to oral PrEP are common occurrences for AGYW which has led to descriptions of AGYW PrEP use as a journey ranging from awareness, initiation, and early use through to persistence, including PrEP pauses, restarts, and discontinuation as their need for PrEP fluctuates with changes in risk behaviors (seasons of risk) [27]. This journey indicates a need for prevention-effective adherence where users take PrEP when they are at risk of HIV acquisition then discontinue when they are no longer exposed to HIV [37, 38]. This is similar to experience in the contraceptive field where women cycle through different patterns of contraceptive use based on partnerships and sexual behavior [39]. There are limited data available on how AGYW’s needs for PrEP change over time and how they recognize when to stop and restart PrEP. It is therefore critical to understand the drivers and correlates of PrEP non-use so we may find ways to better inform efforts to trigger PrEP re-start when it is indicated. With this information, healthcare providers can support end-users to re-start PrEP when there are strong reasons to use PrEP and the protective impact of PrEP can be maximized to ultimately reduce HIV incidence in this young population.

## Reasons for PrEP Non-use/Discontinuation Among AGYW in ESA

Recent literature provides insight into reasons for oral PrEP discontinuation and non-use, including low self-perceived HIV vulnerability or risk of HIV acquisition, side effects related with PrEP use, pill burden/fatigue, disapproval from family and sexual partners, lack of sexual activity, stigma associated with using PrEP, fear of intimate partner violence (IPV), misinformation about drug resistance related to long term oral PrEP use, accidental disclosure, and limited/loss of access [27, 41–45] (Table 2).

**Table 2:** Reasons for AGYW oral PrEP non-use/discontinuation

Study	Countries	Population	Age range (years)	Reasons for PrEP non-use or discontinuation
POWER Qualitative [27]	Kenya (Kisumu), South Africa (Cape Town, Johannesburg)	AGYW (104 in-depth interviews (IDI), 6 focus group discussions (FGD, *n* = 33)	16–25	• Poor perception of HIV vulnerability • Perceived side-effects • Pill-taking burden • PrEP stigma • Disapproval from family and sexual partners
MPYA [46]	Kenya (Thika, Kisumu)	AGYW (50 IDI)	18–24	• Reduction in risky sexual behaviors and lowered risk perception • PrEP fatigue
PrIYA Qualitative [41]	Kenya (Kisumu)	AGYW (93 IDI)	15–24	• Forgetfulness • Difficulty concealing pill taking or swallowing PrEP pills due to size • Limited access • Side effects • Misinformation from male partners that instilled fear about drug resistance if using PrEP on a long-term basis • Male partners confiscating PrEP pills • Male partners subsequently testing HIV-negative • Lack of sexual activity /Time apart from male partners
DREAMS [43]	Kenya (Kisumu)	AGYW (549)	15–24	• Lack of perceived risk • Relocation
SEARCH (Qualitative) [42]	Rural communities in Kenya and Uganda	Young adults (4 male FGDs, *n* = 32 and 4 female FGDs, *n* = 56; 23 IDI)	15–24	• Early side effects of PrEP use • Ending of relationships • Unsupportive partners or needing to hide PrEP use from partners • Being stigmatized by friends • No longer feeling at risk of HIV because of learning their partners’ HIV status • Other life events, such as travel outside the community
HPTN 082/HERS (Qualitative) [44]	South Africa (Cape Town, Johannesburg), Zimbabwe (Harare)	67 AGYW (serial IDIs at 2 time points)	16–25	• HIV stigma where PrEP is mistaken for HIV treatment • Sexual stigma where PrEP was thought to promote sexual promiscuity • Feelings of embarrassment/humiliation about pill bottles being seen or pills heard rattling and resultant teasing
REACH/MTN-034/REACH [45]	South Africa, Zimbabwe, and Uganda	25 AGYW (serial IDIs at 3 time points)	16–21	• Side effects • Large size of the pill making it hard to swallow • Dislike for the taste of the pill • Burden of remembering to take the pill daily • Accidental disclosure through someone discovering the pill bottles, hearing the sound of pills rattling, or seeing the participant taking their pills • Social stigma • Community rumors • School or work schedules conflicting with planned time to take the pills

**Fig. 1:**
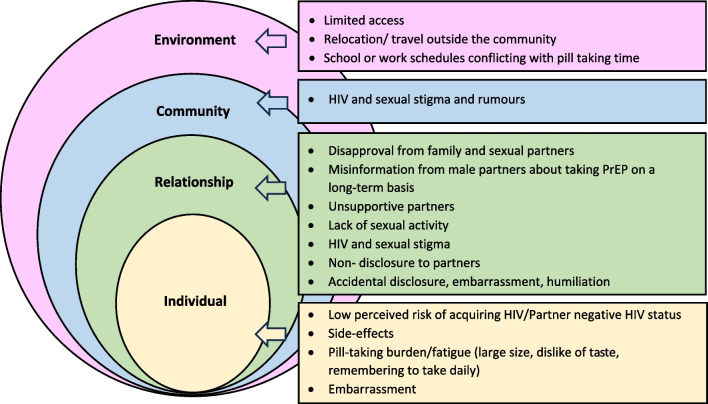
Reasons for AGYW oral PrEP non-use/discontinuation categorized using the socioecological framework

As adolescents exist in a broader context of family, networks and society, these reasons for discontinuation can be imposed over the socioecological framework, a model that emphasizes multiple levels of influence and considers the complex interplay between individual, relationship (social, sexual, family, network), community, and environment [47] (Fig. 1).

From this review, the majority of reasons for discontinuation were related to individual and relationship factors with low perceived risk of HIV acquisition (including poor perception of HIV vulnerability and partner negative HIV status), pill burden, and side effects being frequently cited across many of the studies. Pill burden and side effects are objective challenges that are to be expected given that AGYW are usually healthy and not accustomed to taking medication for long periods or dealing with side effects for a preventive indication. Additionally, as many AGYW live separately from their partners, frequency of sex and potential HIV exposure may be intermittent and daily oral pills (and resulting side effects) for an intermittent risk may be too burdensome. These challenges can be minimized with effective counseling and management of side effects by providers and users. Perception of HIV vulnerability, however, is an individual’s subjective appraisal of the likelihood of being exposed to or acquiring HIV and addressing this factor has potential to assist AGYW recognize periods of heightened HIV acquisition vulnerability in their lives and trigger PrEP restart with sufficient time to ensure protection when necessary.

## Triggering PrEP Restart Among AGYW in ESA

During adolescence, AGYW develop levels of maturity with their growing personal autonomy and are most likely the best advocates and custodians of their sexual health if they are provided with the right tools and the support they need to make informed decisions [48]. As such, AGYW may be empowered to predict and raise self-awareness of their own periods of HIV vulnerability and make decisions to consider using PrEP in ways that match their patterns of possible exposure to HIV. This should include guidance on how to practically assess their actual sexual health vulnerability versus their perceived vulnerability which would then serve as an external cue that they could be exposed to or acquire HIV and should therefore re-start PrEP.

Numerous quantitative HIV risk assessment tools have been developed and are used to identify young women who have demographic, clinical, and behavioral factors that may place them at heightened HIV vulnerability [49–53]. These tools serve several purposes. In community settings, they help counsel women about their periods of HIV vulnerability while in clinical trial settings, they improve recruitment efficiency by targeting enrolment of women who are more vulnerable to HIV. They also assist in the prioritization of these populations for scale-up of new HIV prevention interventions in public health and policy settings through ongoing programs, clinics, and primary care providers [50, 52]. These risk assessment tools could also potentially be used to trigger PrEP restart among AGYW. An analysis of individual-level risk assessment tools used in PrEP demonstration and implementation projects [53] (including 8 studies in ESA with AGYW participants) confirmed that individuals usually have inaccurate and often low perceptions of their own vulnerability to HIV and that accurate vulnerability perception increases with repeated risk assessments through opportunities to reflect on and self-assess personal vulnerability. The majority of tools in the analysis were provider-led and there was a recommendation for the creation of more opportunities for discrete self-administered or combined (self and provider-led) risk assessments (online or otherwise) that are more client/participant centered and put the tool in the PrEP user’s hand for them to internalize and reflect on their own vulnerability for accurate self-awareness. For AGYW, these risk assessment tools should be designed with AGYW input so that it meets their unique needs [47]. One suggestion pertaining to language use when communicating about risk was that the terminology of “being at risk for HIV” be reframed to “vulnerable to HIV” as it may be construed as stigmatizing and act as a barrier to oral PrEP use. Per the article cited, the terminology “vulnerable to HIV” bears less of a negative connotation and includes many things that can make people vulnerable to HIV, including their environment while “risk” tends to blame behavior (as in “taking risks”), with active decisions to engage in unsafe sex, such as sexual violence or unplanned condom-less sex [54]. Further to this, there is also an initiative to reframe and refocus how HIV prevention services for women are developed with a recommendation to move from “HIV risk” terminology to a more empowering “reasons for HIV prevention” based method. Reasons for HIV prevention include personal choices, power, autonomy, and privacy and suggest positive and empowering motivations rather than the negative “risk” and “vulnerability” terms and may help women engage rather than disconnect with their potential HIV prevention needs [55].

In addition to HIV risk assessment tools, the presence of curable STIs such as *Chlamydia trachomatis*, *Neisseria gonorrhoeae* and *Trichomonas vaginalis* also have potential to be used as a marker of heightened HIV vulnerability as there is clear evidence that these STIs increase the risk of HIV transmission [56, 57] and signal personal exposure to a pathogen, most often through condomless sex. AGYW in ESA are subject to high STI prevalence and incidence rates (Table 3). A meta-analysis published in 2018 of over 37,000 women across 18 HIV prevention studies and three primary region/population groups (South Africa community based, Southern/Eastern Africa community-based, and Eastern Africa higher-risk) revealed high prevalence rates of *Chlamydia trachomatis* (estimates ranging from 2.7 to 15.1%), *Neisseria gonorrhoeae* (1.7 to 8.2%), and *Trichomonas vaginalis* (6.7–12.7%) among women aged 15–24 years regardless of region [58]. Individual studies since the review have demonstrated continued high rates. In the Kenya Girls Study, STI incidence was 11% for *Chlamydia trachomatis*, 1.3% for *Neisseria gonorrhoeae*, and 0.8% for *Trichomonas vaginalis* among AGYW aged 16–20 years [59]. Among participants ≤ 24 years enrolled in the ECHO trial conducted in Eswatini, Kenya, South Africa and Zambia sites, 22% and 20% had *Chlamydia trachomatis* at baseline and final visits and 5% and 6% had *Neisseria gonorrhoeae* at baseline and final visits respectively indicating STI persistence or high rates of reinfection, even in clinical trial settings with provision of treatment [60]. In the HPTN082/HERS study, STI incidence was 27.8% per year for *Chlamydia trachomatis*, 11.4% for *Neisseria gonorrhoeae*, and 6.7% for *Trichomonas vaginalis* [61]. The recent MTN-034/REACH study also observed alarmingly high STI prevalence and incidence among younger AGYW (aged 16–21 years) in South Africa, Uganda, and Zimbabwe with 35% testing positive for any STI and 7% having > 1 STI. In this study, 90% of AGYW diagnosed with an STI were asymptomatic [62] and STI incidence was higher among AGYW who were diagnosed with an STI at baseline, despite receiving treatment.

**Table 3:** Summary of STI prevalence and incidence rates in East and Southern African regions

Study	Countries	Duration	No. enrolled	Age (years)	Chlamydia		Gonorrhea		Trichomoniasis	
					Prevalence	Incidence	Prevalence	Incidence	Prevalence	Incidence
Meta-analysis across 18 HIV prevention studies [58]	South Africa, Southern/Eastern Africa	1993–2011	37,000	15–24	2.7 to 15.1% (summary estimate)	-	1.7 to 8.2% (summary estimate)	-	6.7–12.7% (summary estimate)	-
Kenya Girls Study [59]	Kenya (Thika)	2014–2016	400	16–20	11%	-	1.3%	-	0.8%	-
ECHO trial [60]	Eswatini, Kenya, South Africa, and Zambia	Dec 2015 to Oct 2018	4967	≤ 24	22%	20%	5%	6%	-	-
HPTN 082/HERS [61]	South Africa (Cape Town, Johannesburg), Zimbabwe (Harare)	Oct 2016 to Oct 2018	451	16–25	30%	27.8%	7.8%%	11.4%	6.2%	6.7%
MTN-034/REACH [62, 63]	South Africa, Uganda, and Zimbabwe	Jan 2019 to Sep 2021	247	16–21	29%	49.1%	8.5%	21.3%	4.9%	18.8%

The most widely used approach to address STIs in resource limited public healthcare settings like ESA, where laboratory diagnosis is not readily available or accessible, is syndromic management [64]. While less expensive, syndromic management has poor diagnostic accuracy compared to conventional laboratory testing resulting in many STI cases going undetected. More recent STI data show a growing incline in STI prevalence and incidence rates and highlight an urgent need for diagnostic STI management for AGYW. This will ensure targeted and timely STI treatment, inform AGYW perception for HIV and other STI vulnerability, and facilitate data-informed counseling approaches around the HIV and STI syndemics as well as their prevention, including through the use of PrEP. Anecdotal information as well as published qualitative data [65, 66] suggest that AGYW in ESA consider targeted STI testing a benefit over the syndromic management approach and are keen to participate in screening efforts in order to be aware of their STI status. To this end, a variety of point-of-care STI diagnostic tests are being developed or are available for Chlamydia, Gonorrhea, and Trichomoniasis testing with varying assay performance, costs, and resulting time [67]. These point-of-care STI tests have potential to allow individuals to be tested and treated for STIs within the same day thus reducing burden on the individual and the clinic and may be incorporated together with PrEP and contraception services to reduce stigma associated with standalone facilities. Nevertheless, persistent barriers to testing for STIs include fear of positive test result outcomes, stigmatization by parents, family members, public clinic staff and the community, and uncomfortable or embarrassing methods of specimen collection [65]. Self-testing for HIV has been a trail blazer among concepts to support and optimize frequency of testing—and an extension of self-testing to sample self-collection and eventually self-testing for STIs is promising. Uptake, and by proxy, acceptability, of HIV self-testing with either finger-prick blood-based or saliva-based kits among adolescents has been high in initiatives in ESA and has been described as having the potential to revolutionize discrete HIV testing among young people [68–70]. It empowers them to choose the location and timing of the test and control disclosure around their results thus potentially reducing the stigma associated with provider-based testing and offering the convenience of testing external to a public or less private setting. Young people are also attracted by the innovative new technology and have expressed appreciation for the decision-making autonomy and control it gave them at a time of life when they were becoming more independent from their parents and more sexually active [69]. Extending the potential benefits associated with HIV self-testing to other STI would appear the next valuable step in STI management and HIV prevention. While STI self-tests are not yet available, there is potential for AGYW to self-collect vaginal swabs for STI testing based on recent studies showing high rates of acceptability and feasibility of this method due to its privacy, convenience, and time saving nature [71, 72].Self-testing options for common and manageable STIs, such as *Chlamydia trachomatis*, *Neisseria gonorrhoeae*, and *Trichomonas vaginalis* where AGYW perform the test themselves, would appear the next logical innovation needed to facilitate discrete and independent assessment of risk and to trigger PrEP restart.

## Conclusion

AGYW are at the epicenter of the HIV epidemic in ESA and experience evolving challenges with PrEP use and continuation. We found that while reasons for PrEP discontinuation are varied in this population, a common barrier to PrEP persistence was low perceived risk of HIV acquisition. This barrier could be addressed by encouraging accurate self-awareness and recognition of HIV vulnerability, including with behavioral assessment tools and STI self-testing. Innovative and AGYW-tailored interventions, such as self-administered sexual risk assessments and discrete STI testing with effective STI management, are urgently needed. Through these approaches, AGYW can be empowered to make informed decisions about PrEP use that align with their patterns of possible exposure to HIV. Further research to identify and develop these innovations is needed to maximize the impact that available PrEP options can have on HIV incidence in AGYW from ESA.

## Data Availability

Not applicable.
